# Dysregulation of the hypothalamic pituitary adrenal (HPA) axis and physical performance at older ages: An individual participant meta-analysis

**DOI:** 10.1016/j.psyneuen.2012.04.016

**Published:** 2013-01

**Authors:** Michael P. Gardner, Stafford Lightman, Avan Aihie Sayer, Cyrus Cooper, Rachel Cooper, Dorly Deeg, Shah Ebrahim, John Gallacher, Mika Kivimaki, Meena Kumari, Diana Kuh, Richard M. Martin, Geeske Peeters, Yoav Ben-Shlomo, the Halcyon Study Team

**Affiliations:** a School of Social and Community Medicine, University of Bristol, Canynge Hall, Bristol, UK; b Henry Wellcome Laboratories for Integrative Neuroscience and Endocrinology, Bristol, UK; c Medical Research Council Lifecourse Epidemiology Unit, University of Southampton, UK; d MRC Unit for Lifelong Health and Ageing, University College London, UK; e EMGO Institute for Health and Care Research, VU University Medical Center, Amsterdam, The Netherlands; f London School of Hygiene & Tropical Medicine, London, UK; g Department of Primary Care and Public Health, Cardiff University, UK; h Department of Epidemiology and Public Health, University College London, London, UK; i The University of Queensland, Schools of Human Movement Studies and Population Health, Brisbane, Australia

**Keywords:** HPA axis, Physical capability, Healthy ageing

## Abstract

The association between functioning of the hypothalamic pituitary adrenal (HPA) axis and physical performance at older ages remains poorly understood. We carried out meta-analyses to test the hypothesis that dysregulation of the HPA axis, as indexed by patterns of diurnal cortisol release, is associated with worse physical performance. Data from six adult cohorts (ages 50–92 years) were included in a two stage meta-analysis of individual participant data. We analysed each study separately using linear and logistic regression models and then used meta-analytic methods to pool the results. Physical performance outcome measures were walking speed, balance time, chair rise time and grip strength. Exposure measures were morning (serum and salivary) and evening (salivary) cortisol. Total sample sizes in meta-analyses ranged from *n* = 2146 for associations between morning Cortisol Awakening Response and balance to *n* = 8448 for associations between morning cortisol and walking speed. A larger diurnal drop was associated with faster walking speed (standardised coefficient per SD increase 0.052, 95% confidence interval (CI) 0.029, 0.076, *p* < 0.001; age and gender adjusted) and a quicker chair rise time (standardised coefficient per SD increase −0.075, 95% CI −0.116, −0.034, *p* < 0.001; age and gender adjusted). There was little evidence of associations with balance or grip strength. Greater diurnal decline of the HPA axis is associated with better physical performance in later life. This may reflect a causal effect of the HPA axis on performance or that other ageing-related factors are associated with both reduced HPA reactivity and performance.

## 1 Introduction

The hypothalamic pituitary adrenal (HPA) axis is one of several plausible candidate pathways contributing to biological ageing. It activates the secretion of glucocorticoids (cortisol in man) in response to stressful stimuli and exhibits a marked circadian rhythm so that in humans cortisol levels are higher in the morning and decline over the day. A recent report from the Whitehall II (WHII) cohort found that older individuals do have a flatter diurnal pattern (Kumari et al., 2010), a risk factor for age-related chronic conditions, such as cardiovascular disease (Kumari et al., 2011). In the Longitudinal Aging Study Amsterdam (LASA), higher morning and night time cortisol was associated with mortality (Schoorlemmer et al., 2009). Furthermore, a flatter diurnal pattern in older individuals has been associated with poorer cognitive function (O’Hara et al., 2007; Beluche et al., 2010).

Walking speed, standing balance, chair rise time and grip strength are simple, objective measures of physical performance (Birnie et al., 2011; Cooper et al., 2011b). In older populations, sarcopenia (i.e., the loss of muscle mass and strength) (Abellan Van Kan, 2009; Cruz-Jentoft et al., 2010) may develop and there is reduced physical performance, which predicts disability (Guralnik et al., 1995), hospital admissions (Penninx et al., 2000) and mortality (Cooper et al., 2010). The extent to which poor physical performance is linked to age-related changes in regulation of the HPA axis is not well known, but long-term exposure to high cortisol levels, a potent stimulus to protein catabolism, has been postulated as a potential mechanism for sarcopenia (Marcell, 2003). At least three population based studies have reported associations between cortisol levels and physical performance tests (Peeters et al., 2007, 2008; Kumari et al., 2010; Gardner et al., 2011). In the WHII study (Kumari et al., 2010) and Caerphilly Prospective Study (CaPS) (Gardner et al., 2011) individuals with less diurnal variability had slower walking speed, whilst in LASA there were gender differences so that women with higher cortisol levels showed worse performance on a standing balance test and men with higher cortisol levels were slower in walking and chair rises (Peeters et al., 2007). In a longitudinal analysis of LASA, higher cortisol was additionally associated with higher risk of a loss of grip strength over four-years (Peeters et al., 2008).

We have undertaken an individual participant data (IPD) meta-analysis (Riley et al., 2010) from six cohorts, including new unpublished data, as part of the Healthy Ageing across the Life Course (HALCyon) programme, to assess the associations between various measures of cortisol and a range of physical performance tests. This approach has several major advantages: (a) greater statistical power to test for modest associations and sub-group interactions; (b) standardising analyses by grouping covariates in the same way across studies; (c) multiple measures of both cortisol and physical performance allowing a detailed characterisation of the association. We hypothesised that lack of diurnal decline and hence higher cortisol levels across the day would be associated with worse physical performance as this reflects dysregulation of the HPA axis.

## 2 Methods

### 2.1 The cohorts

The Healthy Ageing across the Life Course (HALCyon) research programme is a cross-cohort study on ageing. Of the nine UK cohort studies involved in HALCyon, four have data on both cortisol and physical performance: the Boyd Orr cohort (Martin et al., 2005); the Caerphilly Prospective Study (CaPS) (Smith et al., 2005); the Hertfordshire Cohort Study (HCS) (Syddall et al., 2005); and the MRC National Survey of Health and Development (NSHD) (Wadsworth et al., 2006; Kuh et al., 2011). To increase scientific value (for example enhancing the sample size for individual participant data meta-analysis) we have additionally collaborated with two-large scale cohort studies: the Longitudinal Ageing Study Amsterdam (LASA) (Huisman et al., 2011); and the Whitehall II (WHII) study (Marmot and Brunner, 2005). Details of the cohorts are given in Supplementary Data 1.

### 2.2 Physical performance measures

#### 2.2.1 Chair rises

Chair rising ability was assessed in HCS, LASA and NSHD. In HCS (59–73 years) LASA serum (64–88 years) and LASA salivary (63–92 years), the time taken for participants to stand up from a chair and sit down five consecutive times as fast as possible, was recorded. In NSHD (60–64 years), the same test was undertaken but 10 rises were performed.

#### 2.2.2 Grip strength

Grip strength was assessed in HCS (59–73 years), LASA serum (64–88 years), LASA salivary (63–92 years) and NSHD (age 60–64 years). Dynamometers were used to record either two (LASA) or three (HCS and NSHD) measures in each hand. In HCS and NSHD, maximum grip strength (kg) achieved was used in the main analyses and in LASA grip strength was calculated as the mean of the maximum scores of the left and right hand (kg), as previously published (Peeters et al., 2008).

#### 2.2.3 Standing balance

Standing balance was assessed in Boyd Orr (63–83 years), CaPS (65–83 years), HCS (59–73 years), LASA serum (64–88 years), LASA salivary (63–92 years) and NSHD (age 60–64 years). In Boyd Orr, CaPS, HCS and NSHD, the longest time up to 30 s that a one-legged stand could be maintained with eyes open was recorded. In LASA, the participant stood with one foot behind the other (heel against toe) for 10 s. Two trials were performed in Boyd Orr and CaPS (best values used) and one trial in the other cohorts.

#### 2.2.4 Walking speed

In Boyd Orr (63–83 years), CaPS (65–83 years), HCS (59–73 years) and NSHD (age 60–64 years) the time to get up from a chair, walk 3 m at normal speed, turn around, walking back and sitting down was recorded (timed get up and go) (Podsiadlo and Richardson, 1991). In LASA serum and LASA salivary, the participant walked 3 m along a line, turned around and walked back as fast as possible without running. In WHII (50–73 years), participants walked at a normal pace over a clearly marked 2.4 m course using a standardised protocol (Guralnik et al., 1994) and the time recorded. In LASA, HCS and NSHD one trial was performed, in Boyd Orr and CaPS two trials were performed (average values used) and in WHII three trials were performed (average values used).

#### 2.2.5 Harmonisation of measures

To take account of differences in the protocols used to assess each of the physical performance measures in the different cohorts, the measures were harmonised. Details of the work undertaken to harmonise the measures are provided elsewhere (Cooper et al., 2011a). Firstly, we transformed (log_e_) chair rise times as they were positively skewed. To take account of differences in distances walked in different cohorts we converted walking times into walking speed (metres per minute). In each of the cohorts, balance time was dichotomised at the bottom 20th centile and those in this group were classified as poor balance compared to the rest of the sample. For the purposes of the main analyses, all the outcome measures, with the exception of balance time which had been converted to a binary variable, were then standardised by computing study-specific *z*-scores to further take account of protocol variability.

### 2.3 Cortisol measures

Fasting morning serum cortisol samples were taken, frozen and subsequently measured by radioimmunoassay (Riad-Fahmy et al., 1979) in Boyd Orr (63–83 years) and HCS (59–73 years). In LASA, morning serum cortisol samples were taken at age 64–88 years and the serum levels of cortisol were determined using a competitive immunoassay (Peeters et al., 2007). Both the inter-assay and intra-assay coefficients of variation ranged from 6% in LASA (Peeters et al., 2007) to 8% in HCS.

Salivary cortisol samples were collected in CaPS (65–83 years), NSHD (age 60–64 years), LASA (63–92 years) and WHII (50–73 years). Participants were shown how to collect saliva using plain cotton wool swabs (salivettes) at home. Subjects were requested to chew on the salivettes for 1–2 min and a saliva sample was obtained. In CaPS participants took samples on waking, 30 min later, at 2 pm and 10 pm on two consecutive days. In LASA samples were taken 30 min after waking and at approximately 11 pm. In NSHD samples were taken on waking, 30 min later and at 9 pm. An additional sample was taken at the clinic visit around mid-morning but has not been used in this analysis. In WHII, samples were taken on waking, at waking + 30 min, +2.5 h, +8 h, +12 h and at bedtime. Samples were frozen and subsequently assayed by radioimmunoassay. In CaPS, NSHD and WHII morning salivary cortisol was computed as the mean of waking and 30 min samples. In CaPS, NSHD and WHII assays were done in the same laboratory (Dresden) specialising in high through-put cortisol assays (Kirschbaum and Hellhammer, 1989). In LASA, radioimmunoassay coated tubes (Spectria Orion Diagnostics, Turku, Finland) were used to determine morning and evening salivary cortisol. The inter-assay coefficient of variation for the salivary cortisols ranged from 4% in CaPS at both low (5.3 nmol/l) and high (39.0 nmol/l) controls (Smith et al., 2005) to less than 19% in LASA (Peeters et al., 2007).

### 2.4 Clinical and questionnaire-based data

Anthropometric measures were taken at clinic (Boyd Orr, CaPS, HCS, NSHD and WHII) or by medical interview at home (LASA). Standing height was measured to the nearest mm using a stadiometer (CaPS, HCS, LASA, NSHD and WHII) or a portable measuring stand (Boyd Orr). Weight was measured in kg using a W&T Avery standard model calibrated balance (Boyd Orr), using standardised scales (CaPS and NSHD), a SECA floor scale (HCS and LASA) or by an electronic Soehule scale (Leifheit AS) with a digital readout (WHII). Body Mass Index (BMI) was calculated as weight divided by height^2^ (kg/m^2^). Smoking behaviour was assessed by self-completed questionnaire (Boyd Orr, CaPS and WHII) or medical interview (HCS, LASA and NSHD). The derived variable for smoking status was classified into never, past or current.

### 2.5 Statistics

#### 2.5.1 Missing data

Of those who attended clinic, the percentage with measures of both physical performance and cortisol (physical performance measure given with maximum number of participants when more than one test) was 71% in Boyd Orr, 76% in CaPS, 72% in HCS, 83% in LASA serum, 75% in LASA salivary, 70% in NSHD and 48% in Whitehall II (see Table 1 for sample sizes). In NSHD, salivary cortisol measures were added in the second stage of the data collection, which explains why the numbers are lower than the numbers attending clinic. In Whitehall II, of the 6484 participants who had a clinical assessment, only 4967 were asked to provide a cortisol sample. The lower percentage in Whitehall II was because 1002 cortisol samples were not assayed due to loss of sample in transport between London and Germany or insufficient saliva. This loss of data is likely to be random and not associated with any of the participant characteristics. Those participants excluded due to taking oral corticosteroids are given in Table 1. Only the Whitehall II study was able to exclude morning samples that were not taken within 10 min of waking (Kumari et al., 2010) and the 48% of participants in Whitehall II with both physical performance and cortisol measures are for those that collected samples within 10 min of waking.

#### 2.5.2 Initial treatment of the data and standardisation

Cortisol has a marked circadian rhythm and therefore the time of day at which cortisol is sampled may affect the cortisol level. In CaPS, NSHD and WHII, data on the actual times at which salivary cortisol samples were taken were available. We therefore adjusted the observed values for the time of sampling by fitting a linear or polynomial function to the association between cortisol and time of measurement. In case actual time of sampling predicted cortisol levels, we added residuals from the best fit model to the overall mean cortisol value (Smith et al., 2005). In addition to the morning and night time values, we derived the diurnal drop (difference between morning and evening salivary cortisol samples) and the ‘Cortisol Awakening Response’ (CAR) (difference between the 30 min post waking sample and the waking sample), measures commonly used in epidemiological studies (Adam and Kumari, 2009). In WHII we excluded participants who took samples later than 10 min after waking.

We used linear regression models to analyse chair rise times, grip strength and walking speed and logistic regression analysis for balancing ability. Serum cortisol levels represent total protein bound and free cortisol concentrations in the blood and are around 20 times higher than the free cortisol concentrations found in saliva. A study on the relationship between serum and salivary cortisol in healthy individuals (Reid et al., 1992), showed that correlations were high whether taken at the same time (>0.90) or 70 min apart (0.54–0.94). We therefore converted the absolute levels to study- specific *z*-scores (mean = 0, standard deviation = 1) by dividing the difference between the observed value and the mean by the standard deviation. As night time cortisol was positively skewed, we transformed it (log_e_) before converting it to a *z*-score. We chose potential confounders for the analysis from the literature (age (Larsson et al., 2009), sex (Larsson et al., 2009), adiposity (Larsson et al., 2009) and smoking status (Badrick et al., 2007)) and adjusted the final multivariable model for age (as a continuous term and in quartiles to examine for departure from linearity), sex, Body Mass Index (BMI) (kg/m^2^) and smoking status (never, past or current smoker) at the same time as the cortisol assay.

#### 2.5.3 Analysis

We performed a two stage meta-analysis of individual participant data with each model initially ran within each cohort (the first stage). The cohort-specific effect estimates and standard errors were then pooled using meta-analysis (the second stage). We used fixed effects meta-analysis with the Mantel–Haenszel method except in the presence of significant heterogeneity (*I*^2^ ≥ 35.6%) when random-effects models with the DerSimonian and Laird method were used. These analyses adjusted initially for age and sex and then additionally for BMI and smoking status. We examined potential interactions by running sub-group analyses for sex, age group (above versus below the median age) and adiposity (BMI at ≥30 kg/m^2^ versus <30 kg/m^2^ for obese and non-obese participants). The rationale for these subgroup analysis was that sex differences have been observed previously in LASA, healthy survivor bias may operate in particularly at older ages, and that obese participants may have impaired cortisone (inactive) to cortisol (active) conversion (Stewart et al., 1999).

We undertook a series of sensitivity analyses using: (a) the 30 min post-waking rather than mean morning cortisol level, (b) salivary or serum cortisol from LASA, (c) less than 5 s as the criteria for poor balance, (d) maximum grip strength for the LASA cohort (e) inclusion of subjects with no data due to inability to undertake the test and (f) for HCS using the 3 m walk test rather than the TUG test for walking speed (see Supplementary Data 2).

## 3 Results

Descriptive characteristics of the participants in the six studies are shown in Table 1. Age range was from 50 to 92 years, the youngest cohort being WHII, mean age 61.1 ± 5.9 SD years and the oldest LASA, mean age 74.0 ± 6.1 SD years. Mean walking speed and chair rise times varied considerably between the studies, in part due to differences in tests carried out. Mean chair rise time ranged from 12.7 ± 4.7 SD s for five chair rises in LASA to 24.8 ± 7.3 SD s for ten chair rises in NSHD. Mean walking speed ranged from 34.4 ± 5.5 SD m/min for get up and go test in HCS to 74.1 ± 15.9 SD m/min for the 2.4 m walk test in Whitehall II. For standing balance, the cut-point times for the bottom 20% ranged from 3.7 s in CaPS to 9.1 s in NSHD. Mean grip strength for females ranged from 22.1 ± 6.3 SD kg in LASA to 27.0 ± 7.3 SD kg in NSHD and for males from 35.9 ± 9.0 SD kg in LASA to 45.9 ± 10.9 SD kg in NSHD.

### 3.1 Meta-analyses

**Figure 1:**
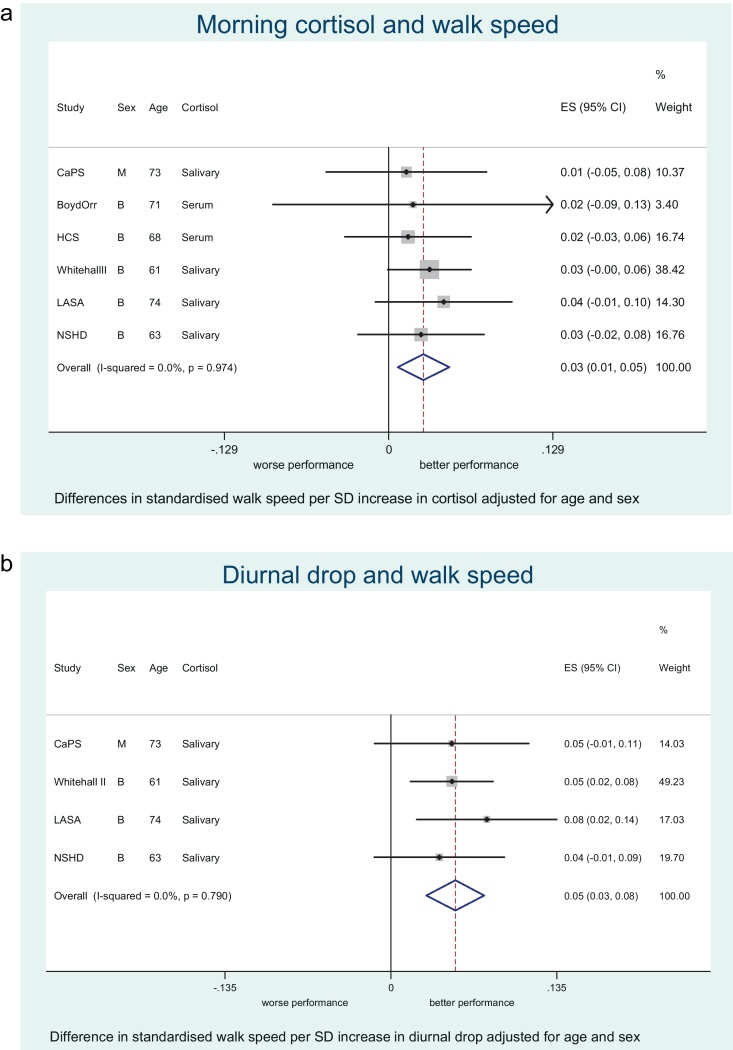
(A) Meta-analysis of the association between morning cortisol and walking speed adjusted for age and gender. (B) Meta-analysis of the association between diurnal drop and walking speed adjusted for age and gender.

Total sample size in the age and sex-adjusted models varied between 2146 and 8448 depending on the meta-analysis (Table 2). Statistically significant summary estimates between the cortisol measures and physical performance from fixed or random effects meta-analyses were as follows: (a) *Walking speed* – higher morning cortisol (Fig. 1A), larger diurnal drop (Fig. 1B) and bigger cortisol awakening response were associated with faster walking speed whilst higher night time cortisol was associated with slower speed in the age and gender adjusted models (all *p*-values < 0.05). After further adjustment for smoking and BMI only the association with morning levels was attenuated; the diurnal drop showed the strongest effect with walking speed. (b) *Chair rises* – higher morning cortisol, larger diurnal drop and lower night time cortisol were associated with quicker chair rise time (note that a positive coefficient indicates worse performance). No association was found with cortisol awakening response, but this result was based on only one study (NSHD). After full adjustment only the association between larger diurnal drop and chair rises was unlikely to be due to chance. (c) *Standing balance* – only a high night time value was associated with worse balance and this association persisted after adjustment for obesity and smoking. There was a suggestive age, sex, BMI and smoking status adjusted association between higher morning cortisol value and better balance (*p* = 0.06). (d) *Grip strength* – no association was observed between cortisol measures and grip strength (see Supplementary Figures for remaining forest plots).

### 3.2 Heterogeneity and sub-group analyses

There was evidence of heterogeneity between studies in age and sex-adjusted meta-analyses of night time cortisol and walking speed (*I*^2^ = 63.6%, *p* = 0.04). In stratified meta-analyses, there was no clear evidence that the associations between the various cortisol measures and physical performance differed by age (above and below the median), obesity (obese or non-obese) or gender. There was weak evidence to suggest that the associations were stronger in non-obese than obese participants for morning cortisol and walking speed but this could have been due to chance (*p* (heterogeneity) = 0.10).

### 3.3 Sensitivity analyses

We found little effect on the meta-analyses for a wide range of sensitivity analyses (see Supplemental Data 2).

## 4 Discussion

The results of these meta-analyses showed that more dynamic activity of the HPA axis, i.e. greater decline, was associated with better physical performance for most of our outcomes, and that there were no interactions with age, gender or BMI. The size of the diurnal decline of cortisol was the strongest correlate of physical performance. Interestingly, all the measures of HPA dynamic activity including higher morning cortisol, lower night time cortisol, greater diurnal drop and increased CAR were all predictive of walking speed although these associations were attenuated to varying degrees after adjustment for smoking and BMI.

A lack of diurnal decline has previously been shown to be associated with increased psychosocial stress (Adam et al., 2006) and a higher BMI (also shown in the current study – data not presented). In WHII (Kumari et al., 2009, 2010), individuals with less diurnal variability had shorter sleep duration and lower sleep quality. The health consequences of a higher morning cortisol are less clear. Some have argued that higher levels may be detrimental and would be associated with adverse outcomes due to increased cortisol exposure. On the other hand, higher morning levels may indicate a more reactive and hence “healthier” HPA axis which should be associated with better health outcomes. We have previously observed in the CaPS (Gardner et al., 2011) that higher morning cortisol was associated with better physical performance, suggesting that the latter explanation may be more valid. The data from this amalgamation of ageing cohorts supports this assertion whereby the ability to demonstrate a good waking response was associated with better performance. Similarly, CAR is larger in healthy individuals (Kudielka and Kirschbaum, 2003) and it has been argued that CAR is an adaptive “boost” response enabling the body to deal with the stresses of the upcoming day (Adam et al., 2006). Thus the observed morning response is complex to interpret as it could reflect the composite effects of increased stress stimulating the HPA axis which could be associated with worse outcomes but this may be counter-balanced by the responsiveness of the HPA axis to a stressor which may be associated with better outcomes.

We failed to find any convincing evidence that any of our cortisol parameters were predictive of grip strength. This is inconsistent with the hypothesis that hypercortisolaemia, as part of the ageing process, may be associated with generalised sarcopenia, but is consistent with the effects seen in Cushing's syndrome where proximal muscle weakness is detected in 56–90% of patients (Fernandez-Rodriguez et al., 2009). We should be cautious before concluding a null association with grip strength: the LASA study found no associations between morning or night time cortisol and grip strength cross-sectionally, but did find that higher cortisol was associated with loss of grip strength over a 4-year period (Peeters et al., 2008). Grip strength is a simple isometric test of upper body strength (Kuh et al., 2005) as compared to walking, chair rising and standing balance which require strength, balance, postural and motor control (Cooper et al., 2010). The dynamic measures of walking speed and chair rising require neuromuscular speed and control (Kuh et al., 2005) and are distinct from balance in that they also require leg muscle power (force × velocity, the ability to generate force quickly) (Peeters et al., 2007). Glucocorticoid mediated steroid myopathy mainly results in type II (fast twitch) fibre atrophy (Fernandez-Rodriguez et al., 2009) which are more efficient for short bursts of speed and power, such as those required for the get up and go or chair rise time tests. These mechanisms may contribute to the stronger associations with walking speed and chair rises than with balance.

We decided to adjust our associations for BMI as a measure of adiposity but whether adiposity is secondary to a less dynamic HPA axis (Bjorntorp, 2001) or vice versa is still unclear (Fernandez-Rodriguez et al., 2009). Obese individuals may have reduced cortisone to cortisol conversion through the expression of 11βHSD1 (Stewart et al., 1999) and this contributes to their lower morning levels and reduced diurnal decline. This may be further accentuated by visceral and subcutaneous adipose tissue 11βHSD1 which shows different circadian rhythmicity with subcutaneous adipose tissue reaching peak gene expression at around 8 am and visceral adipose tissue at around 11 pm (Garaulet et al., 2011). Our BMI-adjusted analyses may be over adjusted if BMI acts as an intermediary in the pathway between cortisol and performance. However, we still observed associations after adjustment though a better measure of visceral adiposity from DEXA imaging may have further attenuated the associations.

A key question is whether the HPA axis is driving physical decline, assuming that our performance measures are reasonable indicators, or whether it is another ageing-related factor impacting on both the HPA axis and physical performance whereby biological ageing drives decline in multiple systems. The associations between cortisol and physical performance in the current study are cross-sectional, hence reverse causation cannot be ruled out. However, similar associations were found prospectively in CaPS when we examined the association between repeat cortisol measures over 20 years and physical performance measures in later life (Gardner et al., 2011).

### 4.1 Strengths and limitations

This is the largest pooled study that has ever looked into the association between cortisol and physical performance and includes new unpublished data from three relatively large studies (Boyd Orr, HCS and NSHD). We undertook an IPD meta-analysis (Riley et al., 2010) which allowed us to standardise analyses in exactly the same way as well as undertake sub-group analyses. A further strength of the study was that we were able to include several objective measures of physical performance from different population-based cohorts. However, we may have still been underpowered for some measures e.g. CAR and for the sub-group analyses and this may have also explained why we did not find many factors which explained the heterogeneity of effect. Possible sources of heterogeneity include the differences in the protocols for assessment of physical performance which might have biased the associations. To overcome such differences we standardised the regression coefficients and dichotomised balance time to make comparisons across cohorts possible, but this may have diluted some effects as we did not examine absolute but rather relative effects. Furthermore, we have undertaken sensitivity analysis using the 3 m walk test in HCS rather than the TUG test and found little effect on the associations. Indeed, other studies (Birnie et al., 2011; Cooper et al., 2011a) have shown that it is possible to combine results from TUG and walking speed tests. Moreover, when more than one trial was performed (Cooper et al., 2011a) it was shown that there was little difference to the findings when analyses were repeated using individual trial, average and best measures.

Finally, there is measurement error in characterising the HPA axis, particularly for those studies that used single serum cortisol measures. Indeed, it has been recommended that the CAR be performed at least twice on two separate days (Hellhammer et al., 2007). The inter-assay coefficient of variation for the salivary cortisol measurements in LASA was high. Assuming random error, this misclassification of true cortisol levels would result in an under-estimation of the true association. More dynamic test such as psychosocial stressors (Kirschbaum et al., 1993) may give more valid results but are difficult to include in large population-based studies.

In conclusion, a more dynamic HPA axis, best measured by diurnal decline, is associated with better physical performance in later life. This may reflect a causal effect of the HPA axis on physical decline or that other ageing-related factors are associated with both reduced HPA reactivity and physical performance. We would recommend that future studies try to collect both morning and night time samples, preferably for several days. We do not think that these associations are driven solely by increased adiposity, though studies with better measures of adiposity would be helpful. In addition, data on sleep quality and duration would allow one to test whether this is a confounding factor (Kumari et al., 2009, 2010).

If the observed associations are causal, diurnal cortisol profile might provide a surrogate intermediary or secondary measures for intervention studies (e.g. weight loss or exercise programmes). Furthermore, diurnal cortisol measures, along with other biological parameters might be used to develop a frailty index (Varadhan et al., 2008) to target interventions to high risk individuals. Further follow-up of these cohorts will enable us to test how cortisol responsiveness changes with age and its predictive effect on ageing traits.

## Ethics

Relevant ethical approval has been received for all studies.

## Role of the funding source

HALCyon is funded by the New Dynamics of Ageing (RES-353-25-0001) and MG and RC are receiving support from this grant. The Boyd Orr cohort has received funding from the Medical Research Council, the World Cancer Research Fund, Research into Ageing, United Kingdom Survivors, the Economic and Social Research Council, the Wellcome Trust and the British Heart Foundation. The Boyd Orr follow-up clinics in 2002 were funded within a Wellcome Research Training Fellowship in Clinical Epidemiology to RMM (GR063779FR). The Caerphilly Prospective Study was undertaken by the former MRC Epidemiology Unit (South Wales) and the School of Social and Community Medicine, University of Bristol acts as the data custodian. The Hertfordshire Cohort Study was funded by the UK Medical Research Council, the Wellcome Trust, Arthritis Research UK and the University of Southampton. The NSHD is funded by the UK Medical Research Council. The WHII study has been supported by grants from the UK Medical Research Council; British Heart Foundation; National Heart Lung and Blood Institute and National Institute on Aging, US, NIH. The Longitudinal Aging Study Amsterdam (LASA) is financially supported by the Dutch Ministry of Public Health, Welfare and Sports. The funders had no role in study design, data collection and analysis, decision to publish, or preparation of the manuscript.

## Conflict of interest

The authors have no conflicts of interest to declare.

## Figures and Tables

**Table 1:** Characteristics of the participants aged 50–94 years by study.

Variable	Boyd Orr	CaPS	HCS	LASA serum	LASA salivary	NSHD	Whitehall II
Clinic attendees^a^	405	1197	2997	1509	1474	2229	6484
Sample with cortisol and physical performance	286	911	2162	1247	1098	1566	3098
Gender (% male)	44.4	100	34.1	52.0	49.5	46.5	74.8
Age (years)	70.5 (4.2)	73.1 (4.0)	67.6 (2.6)	74.0 (6.1)	73.6 (6.5)	63	61.1 (5.9)
Age range at clinic (years)	63–83	65–83	59–73	64–88	63–92	60–64	50–73
BMI (kg/m^2^)	27.4 (4.4)	27.8 (3.8)	27.2 (4.3)	26.7 (3.9)	27.1 (4.1)	27.7 (4.6)	26.7 (4.3)
Smoking status (% yes)	9.8	13.3	9.6	19.3	15.2	14.9	7.9
Serum cortisol (nmol/L)	442.2 (128.9)	–	254.2 (83.8)	491.6 (168.7)	–	–	–
Salivary cortisol (nmol/L)							
Morning	–	19.8 (10.3)	–	–	15.8 (8.0)	23.3 (9.3)	20.0 (8.2)
Night time	–	3.6 (5.2)	–	–	3.9 (4.6)	3.3 (3.8)	2.4 (2.7)
Walk speed^b^ (m/min)	39.2 (8.6)	35.6 (7.6)	34.4 (5.5)	51.3 (14.9)	48.7 (14.5)	42.2 (9.2)	74.1 (15.9)
Standing balance^c^ (s)	17.6 (6.4–30)	15.7 (4.8–30)	19.2 (7.5–30)	10.0	10.0	30.0 (12.3–30)	–
Cut-point (bottom 20%) (s)	4.9	3.7	5.6	9.0	9.0	9.1	–
Chair rise time (s)	–	–	17.4 (4.9)	12.9 (4.5)	12.7 (4.7)	24.8 (7.3)	–
Grip strength (kg)							
Males	–	–	44.6 (7.5)	37.1 (7.2)	35.9 (9.0)	45.9 (10.9)	–
Females	–	–	26.4 (5.6)	22.2 (4.7)	22.1 (6.3)	27.0 (7.3)	–
Exclusions due to oral corticosteroids (%)	0	1.2	0.8	2.1	2.7	0	5.1

a Sample with cortisol and physical performance is the maximum sample size. Results are presented as mean (SD), unless otherwise stated and are based on complete case analysis.

b Walk speed in Boyd Orr, CaPS, NSHD and HCS is the get up and go test. In LASA participant walks 3 m, turns and a further 3 m and in Whitehall II it is a 2.4 m walk. Walk speed was at a normal pace in Boyd Orr, CaPS, HCS, NSHD and Whitehall II and at a fast pace in LASA.

c Standing balance in Boyd Orr, CaPS, HCS and NSHD is a one-legged stand for 30 s, whereas in LASA it is a tandem stand for 10 s. Median balance times (and inter-quartile range) and cut-point times for bottom 20% are given. There is a ceiling effect for the tandem balance in LASA, hence inter-quartile range is not given.

**Table 2:** Overall summary estimates of effect for the associations between cortisol measures and physical performance from fixed or random effects meta-analyses.

Outcome and cortisol measure	Model A (age, sex adjusted)					Model B (age, sex, BMI and smoking status adjusted)				
	ES†	95% CI	*p*-Value	*I*^2^	*p*-Value‡	ES†	95% CI	*p*-Value	*I*^2^	*p*-Value‡
*Walking speed (sd score)*										
Morning (*n* = 8448)	0.027	0.007, 0.048	0.008	0.0%	0.97	0.011	−0.009, 0.031	0.28	0.0%	0.51
Night time (*n* = 6234)	−0.070	−0.113, −0.027	0.001	63.6%	0.041	−0.062	−0.116, −0.007	0.027	78.5%	0.003
Diurnal drop^a^ (*n* = 6063)	0.052	0.029, 0.076	< 0.001	0.0%	0.79	0.041	0.017, 0.064	0.001	0.0%	0.50
CAR^b^ (*n* = 5118)	0.028	0.002, 0.054	0.037	0.0%	0.87	0.031	0.005, 0.057	0.021	0.0%	0.88

*Chair rises (sd score)*										
Morning (*n* = 3441)	−0.037	−0.07, −0.004	0.027	0.0%	0.72	−0.014	−0.047, 0.018	0.38	1.0%	0.36
Night time (*n* = 2307)	0.045	0.005, 0.085	0.027	0.0%	0.75	0.032	−0.007, 0.072	0.11	0.0%	0.35
Diurnal drop^a^ (*n* = 2169)	−0.075	−0.116, −0.034	< 0.001	0.0%	0.48	−0.053	−0.094, −0.013	0.01	23.3%	0.25
CAR^b^ (*n* = 1231)	−0.015	−0.071, 0.040	0.59			−0.019	−0.073, 0.035	0.48		

*Odds of poor balance*										
Morning (*n* = 4570)	1.05	0.94, 1.16	0.41	35.6%	0.18	1.08	1.0, 1.18	0.06	0.0%	0.55
Night time (*n* = 3134)	1.14	1.04, 1.26	0.008	2.8%	0.36	1.12	1.01, 1.24	0.04	32.3%	0.23
Diurnal drop^a^ (*n* = 2985)	1.03	0.89, 1.19	0.69	39.5%	0.19	1.05	0.94, 1.18	0.36	0.0%	0.57
CAR^b^ (*n* = 2146)	1.03	0.92, 1.15	0.59	0.0%	0.90	1.01	0.90, 1.14	0.85	0.0%	0.85

*Grip strength (kg)*										
Morning (*n* = 4781)	−0.056	−0.266, 0.154	0.60	0.0%	0.54	−0.051	−0.264, 0.161	0.64	0.0%	0.41
Night time (*n* = 2382)	−0.274	−0.834, 0.285	0.34	63.1%	0.10	−0.086	−0.757, 0.585	0.80	72.8%	0.06
Diurnal drop^a^ (=2240)	0.117	−0.224, 0.458	0.50	0.0%	0.60	0.052	−0.296, 0.399	0.77	54.6%	0.14
CAR^b^ (*n* = 1224)	0.207	−0.338, 0.752	0.46			0.154	−0.401, 0.708	0.59		

*n* = sample size in age and sex adjusted analyses.

a Diurnal drop is the difference between morning and night time salivary cortisol.

b CAR is the difference between the 30 min post waking sample and the waking sample – for chair rises and grip strength this is only for NSHD. All cortisol measures have been *z*-scored. Random effects meta-analyses were for *I*^2^ ≥ 35.6%, otherwise fixed effect meta-analyses were used.

† Mean difference in standardised walking speed; mean differences in standardised chair rise time (log_e_); Odds Ratio of poor balance (lowest 20%); mean difference in grip strength.

‡ *p*-Value is obtained from the heterogeneity *χ*^2^.
