# Supplementary material for: Dysregulation of the hypothalamic pituitary adrenal (HPA) axis and physical performance at older ages: An individual participant meta-analysis
Source: Psychoneuroendocrinology. 2013 Jan;38(1):40–9. doi: 10.1016/j.psyneuen.2012.04.016 (PMC3533133; doi:10.1016/j.psyneuen.2012.04.016)
Supplement: Supplementary file 1 [file mmc1.docx]

**Supplementary Data 1**

**Cohorts**

*Boyd Orr Cohort*

The Boyd Orr study is an historic cohort of men and women born between 1918 and 1939 who participated in the Carnegie (Boyd Orr) Survey of Diet and Health in Pre-War Britain, 1937-1939 (1). In 2002, 732 surviving study members living in or near Bristol, London, Wisbech, Aberdeen and Dundee and who had previously consented to follow-up were contacted and 405 participants subsequently took part in a detailed clinical examination to obtain physiological measurements and blood assays when aged 63-83 years.

*Caerphilly Prospective Study (CaPS)*

CaPS is a cohort of men born between 1920 and 1939 who were recruited between 1979 and 1983 from Caerphilly and adjacent villages. 2512 men (response rate of 89%) were seen when they were 45-59 years and were followed up at phase 2 (1984-1988), phase 3 (1989-1993), phase 4 (1993-1996) and phase 5 (2002-2004). At phase 2, an additional 447 men of similar age were recruited who had moved into the defined area. Cortisol and physical performance measures undertaken at phase 5 are those included in the current study.

*Hertfordshire Cohort Study (HCS)*

HCS is a cohort of men and women born in Hertfordshire (East, North or West) between 1931 and 1939 whose detailed birth and infant records were available and who were alive and still living in Hertfordshire in the 1990s. Wave 1 data for HCS were collected in 1999-2004 when study participants were aged 59-73y. 2997 participants attended the clinic at wave 1. Cortisol and physical performance measures undertaken at wave 1 are those included in the current study.

*MRC National Survey of Health and Development (NSHD)*

NSHD is the 1946 British birth cohort and is a representative sample of people born in England, Scotland and Wales during one week in March 1946 who have been followed up prospectively since birth. 1880 participants had saliva collected when they were aged 60-64 years. The cortisol and physical performance measures that we have used come from the examination when the participants were aged 60-64 years. Saliva samples for cortisol assessment were collected in 2006/2009 and processed in 2009/2011.

*Longitudinal Ageing Study Amsterdam (LASA)*

The Longitudinal Ageing Study Amsterdam (LASA) is a cohort study on predictors and consequences of changes in physical, cognitive, emotional and social functioning in older persons. Baseline measurements of LASA were undertaken in 1992-1993 when participants were aged 55-85 years and have been followed up at cycle 2 (1995-1996), cycle 3 (1998-1999), cycle 4 (2001-2002), cycle 5 (2005-2006) and cycle 6 (2008-2009). 1509 participants had blood collected at cycle 2 and 1474 participants had saliva collected at cycle 4. Cortisol and physical performance measures are available at cycle 2 and cycle 4. Serum was stored at -70^0^C until processing in 2002/2003.

*Whitehall II Study*

Whitehall II is a cohort of men and women initially recruited between 1985 and 1988 (phase 1) from 20 London-based civil service departments when participants were aged 35-55 years. At phase 1, 10,308 people participated and eight phases of the study have been completed. 6484 participants had a clinical assessment at phase 7. Cortisol and physical performance measures undertaken at phase 7 (2002-2004) are those included in the current study.
