# Supplementary material for: Dysregulation of the hypothalamic pituitary adrenal (HPA) axis and physical performance at older ages: An individual participant meta-analysis
Source: Psychoneuroendocrinology. 2013 Jan;38(1):40–9. doi: 10.1016/j.psyneuen.2012.04.016 (PMC3533133; doi:10.1016/j.psyneuen.2012.04.016)
Supplement: Supplementary file 2 [file mmc2.docx]

**Supplementary Data 2**

**Methods-Sensitivity analysis**

As well as using the mean of waking and 30 minutes post waking sample as the morning cortisol sample in CaPS, NSHD and Whitehall II, we also repeated analyses but using the 30 minutes post waking sample as morning cortisol in these cohorts. This is similar to the morning cortisol measures in Boyd Orr, HCS and LASA. We also carried out a sensitivity analysis using the 30 minutes post waking cortisol as the anchor for the diurnal drop. LASA had both salivary and serum morning cortisol measures, but only salivary night time cortisol measures. As the main analyses were undertaken using salivary cortisol from LASA (this enabled analysis of diurnal drop), we repeated meta-analyses for associations between morning cortisol and physical performance but using serum cortisol from LASA. As balance time was dichotomised at the bottom 20^th^ centile, we undertook sensitivity analysis to take into account the variation in age of this measure for the cohorts with a wide age range. We did this by creating age-specific 20% cut-points for balance time and repeated meta-analyses for associations between cortisol measures and balance. In LASA, participants balanced for up to 10 seconds, whilst in the other cohorts participants balanced for up to 30 seconds. We therefore repeated meta-analyses for associations between cortisol measures and balance but this time dichotomising balance below or above 5 seconds. In LASA, we undertook a sensitivity analysis using maximum grip strength (Kg) achieved in the main analysis. Some participants were unable to perform the tests because of surgery, injury, other health problems or unwillingness and had missing data. We repeated the meta-analyses with standing balance and walking speed by classifying these participants as having a random value of physical performance within the bottom 20% of the distribution. We repeated the meta-analyses for walking speed for HCS but including the standard 3m walk test at normal pace, rather than the timed get up and go test (TUG).

**Results- Sensitivity analysis**

Treating age as a categorical variable (quartiles) rather than a continuous variable had little effect on the associations in multivariable models (data not shown). We found little difference to the associations when using 30 minutes post waking as the morning sample and as anchor in the diurnal drop (data not shown). Furthermore, there was little effect on the meta-analyses for associations between morning cortisol and physical performance whether serum or salivary cortisol was measured in LASA (data not shown). Creating age-specific 20% cut-points for balance made little difference to the meta-analyses (data not shown). Dichotomising balance below or above 5 seconds rather than classifying poor balance in the bottom 20% of the distribution made little difference to the meta-analyses (data not shown). Using maximum grip strength in LASA, rather than the mean of the maximum scores of the left and right hand made little difference to the results (data not shown). The overall percentage of participants unable to perform the tests across cohorts was 9.5% for the balance test and 3.4% for walking speed. Including the participants who could not complete the balance or walking speed tests made little difference to the results (data not shown). Using the standard 3m walk test rather than the TUG test in HCS made little difference to the associations (data not shown).
