# Supplementary material for: Dysregulation of the hypothalamic pituitary adrenal (HPA) axis and physical performance at older ages: An individual participant meta-analysis
Source: Psychoneuroendocrinology. 2013 Jan;38(1):40–9. doi: 10.1016/j.psyneuen.2012.04.016 (PMC3533133; doi:10.1016/j.psyneuen.2012.04.016)
Supplement: Supplementary file 3 [file mmc3.docx]

**Supplementary Figures**

**Figure s1** Meta-analyses of the associations between night time cortisol and walking speed (A) and CAR and walking speed (B) adjusted for age and gender

**Figure s2** Meta-analyses of the associations between morning cortisol and chair rise time (A), night time cortisol and chair rise time (B) and diurnal drop and chair rise time (C) adjusted for age and gender

**Figure s3** Meta-analyses of the associations between morning cortisol and balance (A), night time cortisol and balance (B), diurnal drop and balance (C) and CAR and balance (D) adjusted for age and gender

**Figure s4** Meta-analyses of the associations between morning cortisol and grip strength (A), night time cortisol and grip strength (B) and diurnal drop and grip strength (C) adjusted for age and gender

**Figure s1A**

**Figure s1B**

**Figure s2A**

**Figure s2B**

**Figure s2C**

**Figure s3A**

**Figure s3B**

**Figure s3C**

**Figure s3D**

**Figure s4A**

**Figure s4B**

**Figure s4C**
